# A novel technique for sacropelvic fixation using image-guided sacroiliac screws: a case series and biomechanical study

**DOI:** 10.7555/JBR.32.20170077

**Published:** 2018-06-15

**Authors:** Kee D. Kim, Huy Duong, Aditya Muzumdar, Mir Hussain, Mark Moldavsky, Bandon Bucklen

**Affiliations:** 1. Department of Neurological Surgery, University of California, Davis, Sacramneto, CA 95817, USA; 2. Department of Neurosurgery, Kaiser Permanente Medical Group, Sacramento, CA 95825, USA; 3. Musculoskeletal Education and Research Center (MERC), Globus Medical Inc, Audubon, PA 19403, USA.

**Keywords:** lumbosacral fixation, sacropelvic fixation, sacroiliac screw, computer-assisted surgery, image-guided surgery

## Abstract

In this study, we sought to assess the safety and accuracy of sacropelvic fixation performed with image-guided sacroiliac screw placement using postoperative computed tomography and X-rays. The sacroiliac screws were placed with navigation in five patients. Intact specimens were mounted onto a six-degrees-of-freedom spine motion simulator. Long lumbosacral constructs using bilateral sacroiliac screws and bilateral S1 pedicle and iliac screws were tested in seven cadaveric spines. Nine sacroiliac screws were well-placed under an image guidance system (IGS); one was placed poorly without IGS with no symptoms. Both fixation techniques significantly reduced range of motion (*P*<0.05) at L5–S1. The research concluded that rigid lumbosacral fixation can be achieved with sacroiliac screws, and image guidance improves its safety and accuracy. This new technique of image-guided sacroiliac screw insertion should prove useful in many types of fusion to the sacrum, particularly for patients with poor bone quality, complicated anatomy, infection, previous failed fusion and iliac harvesting.

## Introduction

Posterior lumbosacral instrumented fusion is widely used to treat spinal instability caused by a variety of conditions including degenerative disease, infection, tumor, trauma and deformity^[1^–^3]^. When the instrumented construct is long and extends past the lumbar region, a large moment arm is placed on the caudal end, putting the construct at risk for fusion failure, instrumentation pullout, sacral fracture and loss of lordosis^[4^–^5]^. The construct will be in additional jeopardy when the spine is osteopenic or osteoporotic, and post-radiation.

One commonly used approach to strengthen the caudal end of lumbosacral constructs is to extend fixation to the pelvis with the use of bilateral iliac screws. They are placed with bicortical purchase in an anterolateral direction from the posterior superior iliac spine into the hard cortical bone above the sciatic notch, thereby resisting the cantilever moment of the long construct at the cranial end^[2^,^6]^. The iliac screw heads are generally positioned lateral to the axis of lumbar/sacral screws, and therefore require additional bending of the rods, or the use of an offset connector. In this article, we present an alternative technique for strengthening the lumbosacral construct using bilateral sacroiliac screws. The sacroiliac screws are also inserted in the anterolateral direction into the hard cortical bone above the sciatic notch to optimize resistance against the cantilever moment of a long construct superiorly. The heads of the sacroiliac screws are in-line with the rest of the lumbosacral screws in a long construct, which obviates the need for additional bending of the rod or the use of offset connector.

In this study, we evaluated the biomechanical parameters of sacroiliac I screws in cadavers with nondestructive range of motion (ROM) and flexibility testing. We present the results of our study, and will discuss the advantages of sacroiliac screws over traditional iliac screws. We will also describe the technique of sacroiliac screw placement with use of image guidance/fluoroscopy to make sacropelvic fixation safer and more accurate..

## Materials and methods

Sacroiliac screws were placed in five patients under image guidance from 2005 through 2007 with permission from, and in accordance with the guidelines of, the Institute of Research and Ethics (*Table 1*). All patients were referred to the senior author (KDK) through the UC Davis Health System. The decision to perform sacropelvic fixation was mainly based on the need for additional points of fixation in the pelvis to attain successful lumbosacral fusion. All patients had received preoperative lumbosacral CT and MRI scans — and, in some cases, plain radiographs.

**Tab.1:** Characteristics of five consecutive cases of image-guided sacropelvic fixation performed by senior author from 2005 through 2007

Case No.	Age/Gender	Indications for surgery	Procedure
1	29/M	Trauma: S1–S2 sacral fracture w/cauda equina syndrome	S1–S3 decompression with L4–S3 fusion using bilateral S2/ sacroiliac screws and S1 and S3 pedicle screws
2	63/M	Infection/Reoperation: L4–L5 osteomyelitis fracture/dislocation	Removal of old screws and circumferential decompression and fusion using L1–S1 pedicle and S2/ sacroiliac screws
3	84/F	Degeneration/Reoperation: Lumbar scoliosis and stenosis with three prior surgeries and osteoporosis	L2–L5 decompression and T12–S2 fusion using S1 pedicle and bilateral S2/ sacroiliac screws
4	58/F	Degeneration: Lumbar stenosis and scoliosis	L2–S1 decompression fusion using T12–S1 pedicle and S2/ sacroiliac screws
5	54/M	Infection/Reoperation: Chronic osteomyelitis after prior surgeries	Removal of anterior instrumentation and corpectomies T10–L3 with T9–L4 cage. Staged T7–T10 and L4–L5 pedicle screws with bilateral S1/ sacroiliac screws

### Image guidance and surgical procedure

**Fig.1:**
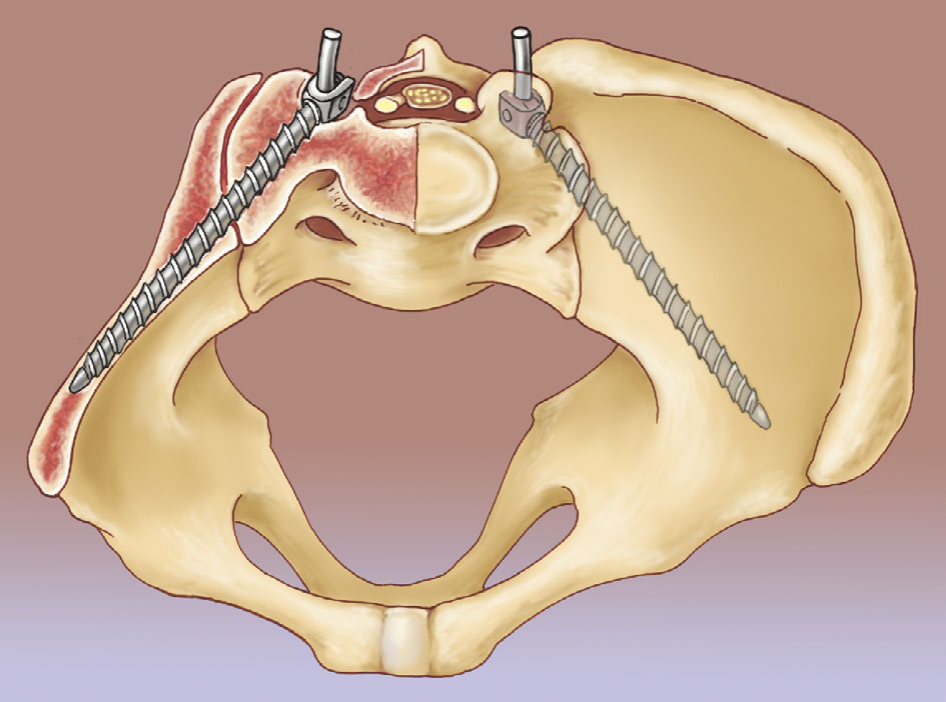
Artist’s rendering of the pelvis showing sacroiliac screw position.

**Fig.2:**
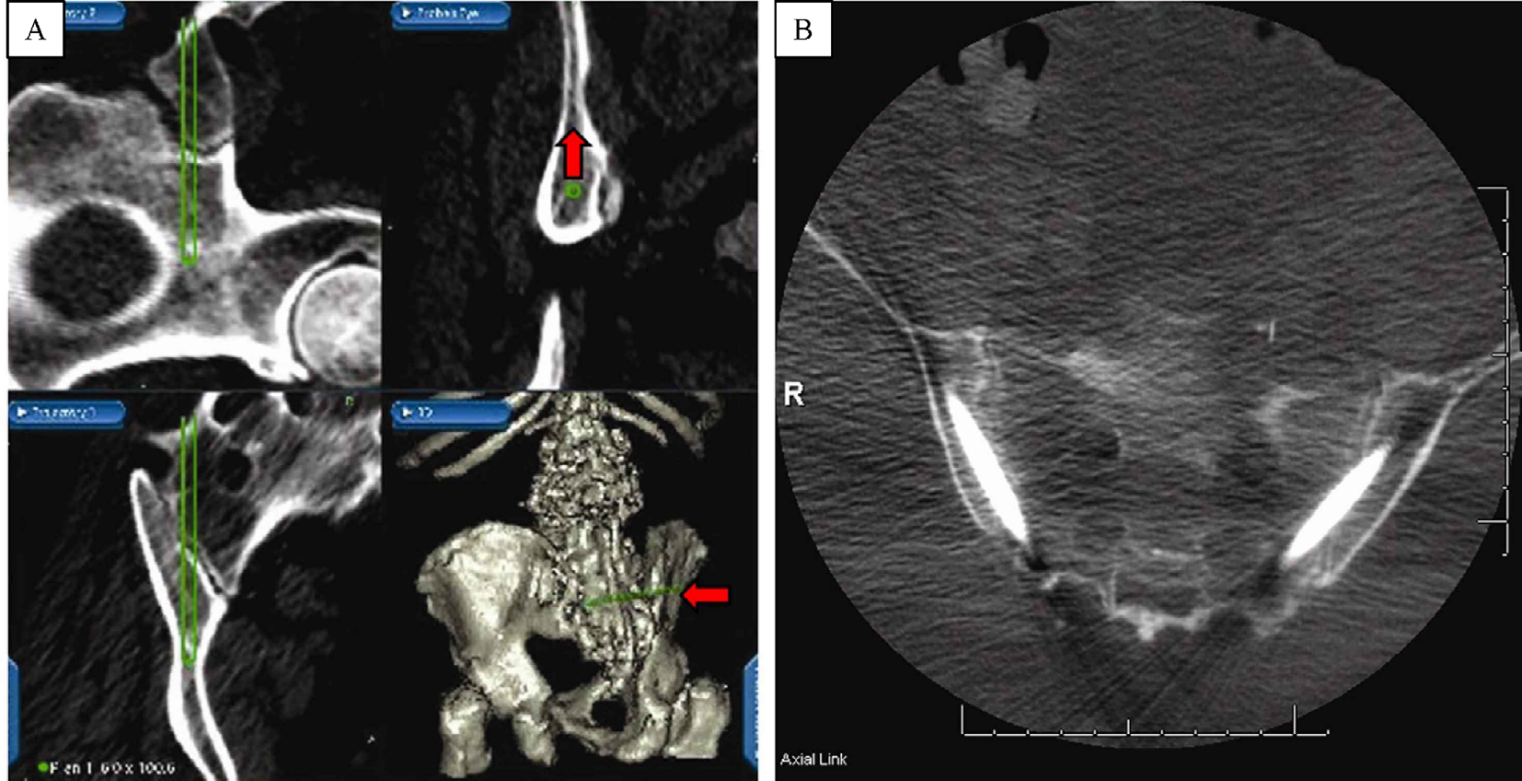
Stealth IGS monitor snapshot. A: Upper left: parasagittal view showing the trajectory from the sacrum to the pelvis. Upper right: probe’s eye view of the ilium. This view is particularly helpful for planning a screw trajectory that avoids cortical breech and has an ideal location, where a screw may be surrounded by stronger cortical bone (arrow). Lower left: the axial view of sacropelvic screw trajectory. Lower right: 3D view of the sacropelvic region. Note the extensive iliac defect (arrow) on the right from an iliac crest harvest performed during previous surgery. Image guidance is helpful in planning the trajectory to avoid the area of defect/weakness. B: Postoperative axial CT showing good placement of bilateral sacroiliac screws.

The Stealth Station Image Guidance System (IGS) (Medtronic Navigation, Louisville, CO, USA) was used for preoperative trajectory planning and intraoperative screw placement. Preoperative CT data were imported into the IGS computer workstation, and anatomic registration points were selected. The points selected on bony landmarks were those that were most likely to be identified once standard posterior lumbosacral exposure for posterolateral fusion was attained. Registration landmarks usually consisted of one or two spinous processes, and two to four facet joints in the lumbosacral region. In patients undergoing reoperation, any easily identifiable bony landmarks were chosen. The ideal screw trajectory traversed the cortices of the sacroiliac joint, with the tip of the screw within the ilium in the cortical bone above the sciatic notch (*Fig. 1*). Sacroiliac screw trajectories were planned using two-dimensional navigational and three-dimensional (3D) reconstructed views (*Fig. 2*). Maximum ideal screw length and diameter were determined from the preoperative virtual plan.

A midline skin incision and subperiosteal dissection were performed to expose the relevant spinal levels out to the transverse processes and the sacral ala. A passive reference arc was clamped onto the spinous process of the most cephalad vertebra that was exposed. The reference arc was cranially angled away from the lumbosacral region to minimize the possibility that it could be inadvertently moved after registration. Point registration and surface merging were completed to optimize accurate registration. Good registration accuracy was consistently verified by touching observable bony landmarks with the IGS probe tip and correlating probe location, with the virtual image displayed on the monitor. Pedicle screws were typically inserted at lumbar levels using standard techniques.

Next, in keeping with the preoperative plan, IGS was used to select sacroiliac screw entry points on the sacrum. A new entry point not included in the preoperative plan may have been selected to facilitate rod alignment, contouring and attachment to screw heads. Typically, the point of entry was noted at S1 or S2. The cortical wall of the sacrum was penetrated by an IGS awl. Next, an IGS sounding probe was used to create a pilot hole for the screw. The pilot hole trajectory may have been modified from the virtual plan for placement of the screw in the ideal portion of the ilium (the cortical bone above the sciatic notch). The sounding probe was advanced with IGS tracking. The virtual navigation view was used with tactile feedback to discern when the sacroiliac joint was being traversed. Pilot hole integrity was checked with a ball-tip probe; then, the IGS tap was used to tap the same pilot hole. The tapped hole was typically probed again to assess for cortical breach. Sacroiliac screws were then inserted, with care taken to avoid deviating from the tapped trajectory. Sacroiliac screws were connected to the remaining pedicle screws with contoured rods to form a physiologic lumbosacral curve. The fusion surface was routinely decorticated, and bone graft was placed after the rod had been secured with setscrews. A postoperative CT scan was performed to check the screw position.

### Biomechanical testing

**Fig.3:**
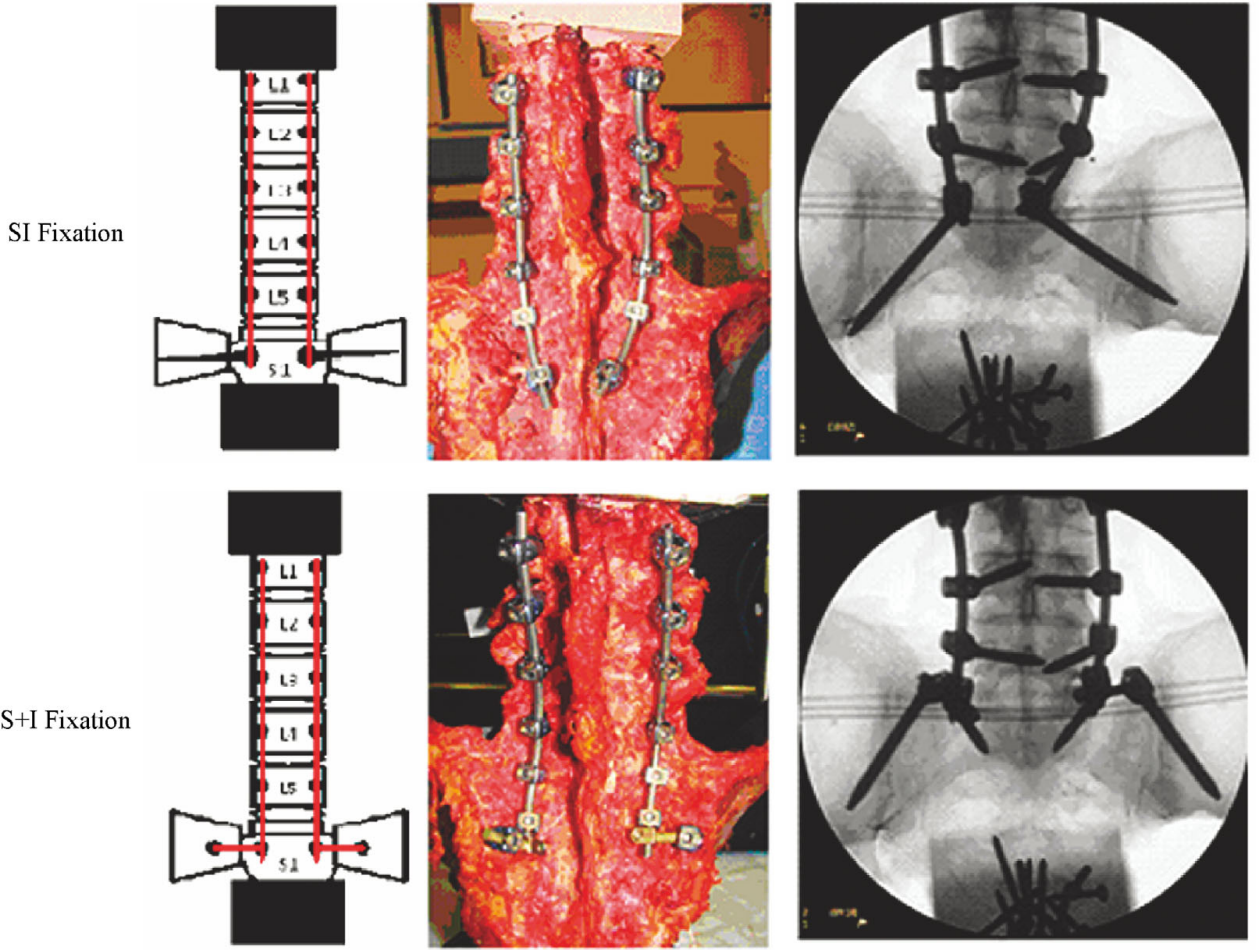
Diagrams, photographs and radiographs with the two surgical constructs (Sacroiliac fixation *vs*. bilateral S1 pedicle screw and iliac screw [S+I] fixation). SI: sacroiliac.

Seven cadaveric spines (ilio-lumbosacral) were harvested and stripped of all musculature while all of their ligamentous structures, vertebral bodies and intervertebral discs were kept intact. X-rays were obtained to assess the specimens’ bone quality. Spine specimens were secured into test fixtures at L1 and the sacrum. Intact specimens were mounted onto a six-degrees-of-freedom spine motion simulator, and nondestructive ROM flexibility testing was conducted by applying pure moments (±6 Nm) in three randomly selected physiologic planes: flexion-extension, lateral bending and axial rotation^[7^–^8]^. Segmental L5–S1 motions were collected via an optoelectronic motion measurement system with infrared light-emitting diodes placed at L5 and S1. After intact testing, 6.5-mm-diameter bilateral pedicle screws (REVERE^®^ Stabilization System, Globus Medical, Inc., Audubon, PA, USA) were placed at L1–L5, without violation of facet joints or disc spaces. Iliac screws that were 7.5 mm in diameter were used, and a 5.5-mm-diameter rod was instrumented for all biomechanical testing to be performed. Sacroiliac fixation was performed by using (1) bilateral sacroiliac screws and (2) bilateral S1 pedicle screws and iliac screws (S+I) (*Fig. 3*). Lumbosacral rods were sized and contoured appropriately for the seven specimens. Additional offset connectors were used to connect iliac screws to rods (S+I construct). ROM data were normalized relative to the value of the intact spine. Statistical analysis was performed on the raw data using repeated measures analysis of variance and Tukey’s post hoc test (*P*<0.05). The Data Analysis Tool Pack available in Microsoft Excel (Microsoft Corp., Redmond, WA, United States) was used to run the analysis of variance, and the post-hoc test was calculated based on the equation for Tukey’s honest significant difference test.

## Results

### *In vivo* sacroiliac screw placement

**Fig.4:**
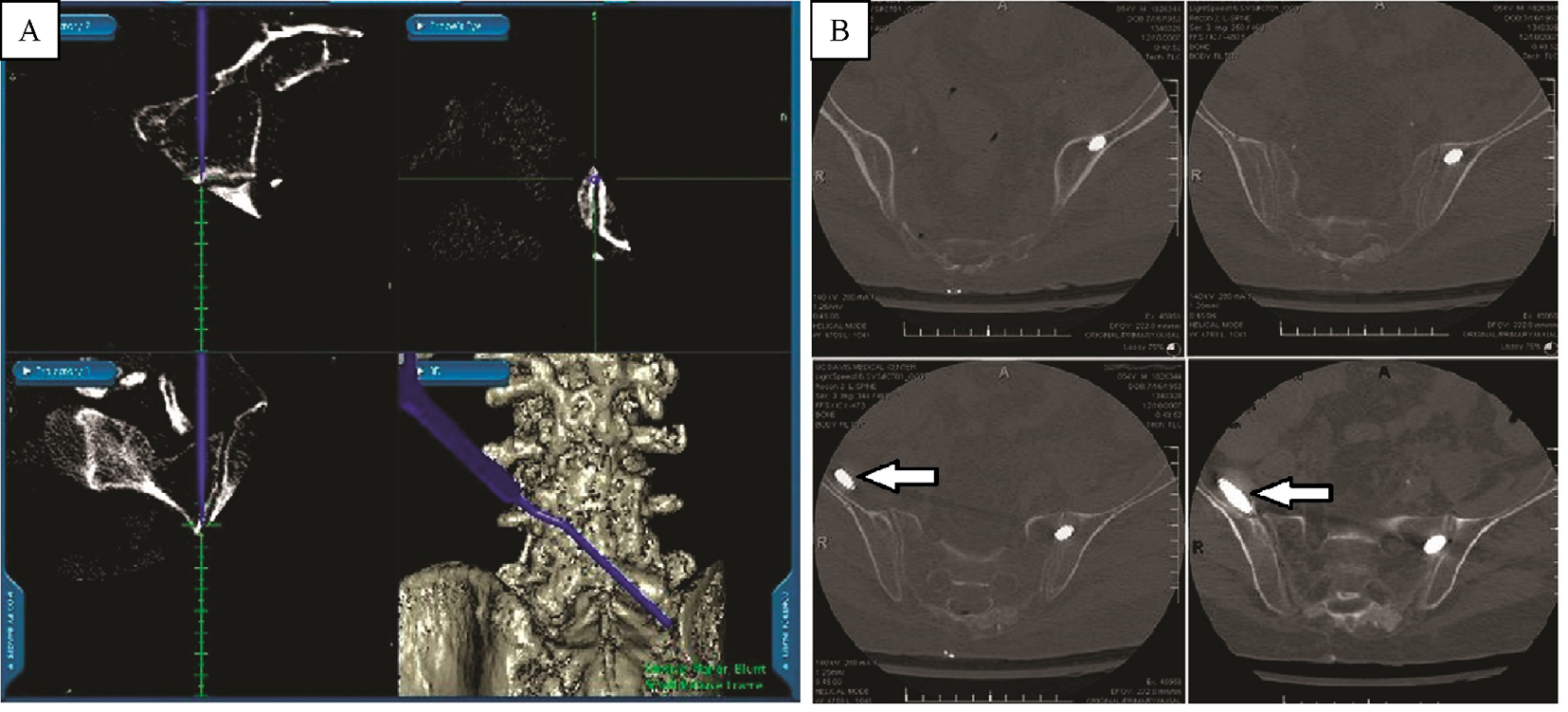
Biomechanial study constructs. A: Stealth IGS snapshot showing preoperative trajectory planning. Note the lack of inclusion of the right ilium in the CT field of view. B: Postoperative CTshowing axial images of malpositioned right SI screw placed under fluoroscopic guidance (indicated by arrows) compared with a properly positioned left sacroiliac screw placed via IGS. IGS: image guidance system.

Five patients (four males, and one female) with a mean age of 58 years (range: 29–84 years) underwent screw placement under an IGS to allow visualization of the screw trajectory (*Table 1*). In all cases, the surgical indication for placement of sacroiliac screws was the need to supplement a planned lumbosacral fusion with sacropelvic fixation to add strength to the construct. In two cases (*Table 1,* Cases 2 and 5), anterior instrumented fusion was combined with posterior fusion. Diagnoses of treated patients included severe degenerative disease (*n* = 2), infection (*n* = 2) and trauma (*n* = 1). A total of nine sacroiliac screws were placed under IGS, and one without. Eight sacroiliac screws were placed with entry points in S2, and two were placed with entry points at the caudal aspect of S1 (*Table 1,* Case 5). All image-guided screws were placed in good anatomic positions. The right-sided screw in Case 5 was placed without image guidance, as the field of view on the preoperative CT scan did not include the ilium on the right side. Postoperative CT showed that this screw was malpositioned with ventral perforation into the retroperitoneal space adjacent to the iliopsoas (*Fig. 4*), but the contralateral image-guided screw position was ideal. In the present series, no vascular or neurologic injuries were detected postoperatively. Follow-up at longer than 2 years revealed no construct failures. During the first postoperative month, one patient (*Table 1, Case 5*) died from complications of aspiration and sepsis not related to the surgery.

### *In vivo* comparisons with intact

During flexion-extension, ROM for sacroiliac and S+I constructs was (31.1±16.7)% and (35.9±14.9)% respectively, compared with the intact. In lateral bending, SI and S+I constructs reduced motion to (37.3±17.5)% and (38.5±17.4)%, respectively, compared with the intact. Finally, during axial rotation, sacroiliac and S+I constructs reduced ROM to (77.6±21.3)% and (72.6±23.0)%, respectively, compared with the intact.

### *In vivo* range of motion

**Fig.5:**
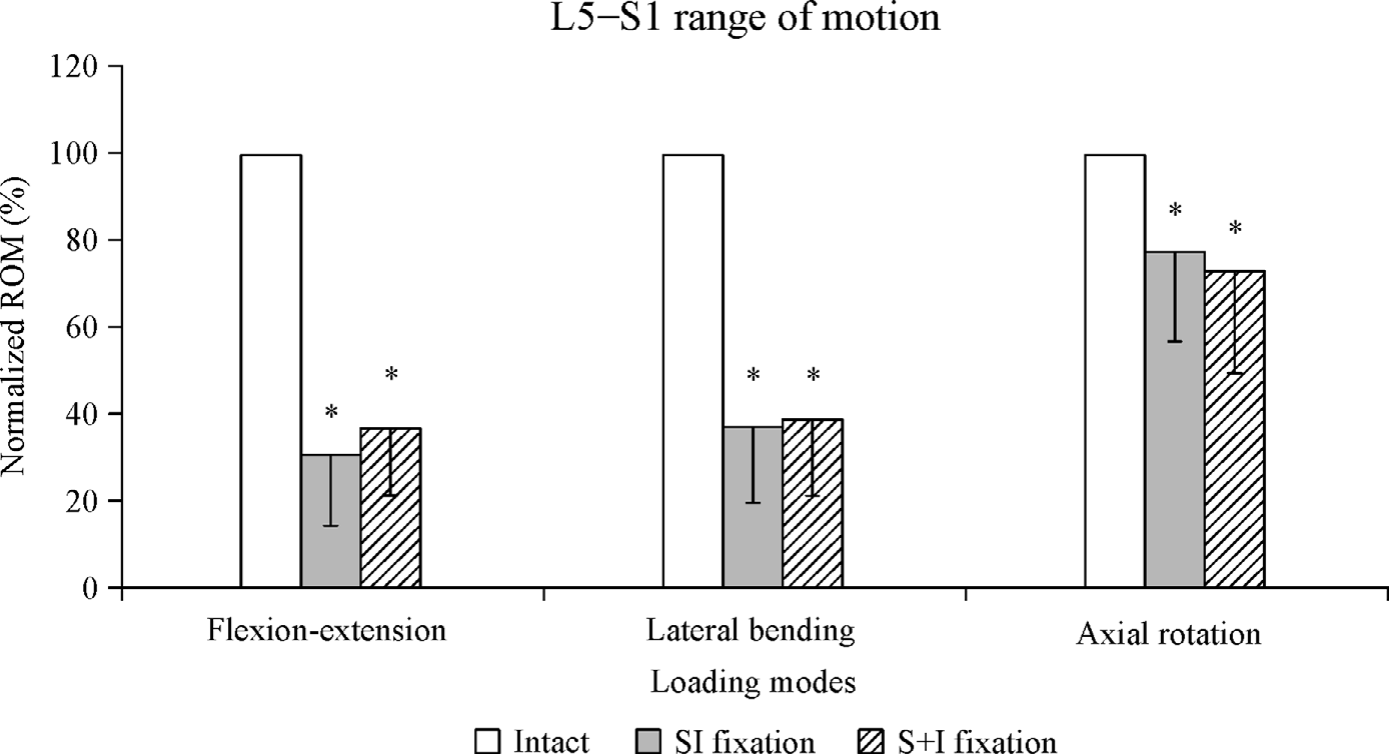
Normalized ROM (%) at L5–S1 for all loading modes. ROM: range of motion. * P < 0.05 vs. intact.

Both fixation techniques significantly reduced ROM (*P*<0.05) at L5–S1 compared with the intact in all loading modes (flexion-extension, lateral bending and axial rotation; *Fig. 5*). We observed no statistically significant differences (*P*<0.05) in the stability offered by both techniques in any of the loading modes. ROM with sacroiliac fixation showed a slight decrease in flexion-extension and lateral bending, as well as a slight increase in axial rotation in comparison to S+I fixation.

## Discussion

Various sacral screws, and iliac bolts or screws, have been utilized for lumbosacral constructs^[9]^. Orthopaedic surgeons have described the percutaneous placement of iliosacral screws inserted from the ilium to the sacrum for pelvic ring reconstruction, including the use of imaged-guided fluoroscopy^[10^–^15]^. To our knowledge, this is the first report in English literature that describes the use of image guidance for a long screw with an entry point in the sacrum, traversing the SI joint and terminating within the ilium.

### Rationale

Pseudoarthrosis rates as high as 45%, with in situ fusion of the lumbosacral junction, have been reported for high-grade spondylolistheses^[16]^. Achieving a solid arthrodesis is made more difficult by additional challenges posed by prior surgery, infection, osteoporosis or severe deformity. Consequently, various augmentation techniques for lumbosacral arthrodesis have been reported^[6^,^16]^. One option for increasing the rate of solid fusion involves using an interbody graft at the L5–S1 disc space^[17^–^18]^.

Another option for augmenting lumbosacral fusion involves using the ilium to share the stress and load placed on the sacrum. Iliac screw fixation has been shown to improve biomechanical strength at the lumbosacral junction, with reasonable clinical outcomes^[6^,^19]^. In comparison to other methods of sacral fixation such as intrasacral rods, iliac screws are more effective in preventing S1 pedicle screw failure^[6^,^9^,^18]^. The additional points of fixation attained with iliac screws provide greater protection than what is provided by interbody arthrodesis with posterior instrumentation at L5–S1 alone^[17]^.

Researchers have asserted that the best available construct for minimizing lumbosacral instrumentation failure and enhancing fusion consists of long, bilateral iliac screws and bicortical sacral screws with anterior column support. Iliac screws frequently require removal due to the discomfort associated with their prominence over the posterior iliac wing at the PSIS^[6^,^20]^. Sacroiliac screws, on the other hand, are no more prominent than sacral pedicle screws.

Iliac screws (in the bilateral S1 pedicle screw and iliac screw [S+I] construct) introduce the disadvantage of awkward attachments to the lumbosacral rods via short offset connectors, contributing to the potential for mechanical failure at this additional interface. Alternatively, sacroiliac screws can be aligned readily with lumbosacral screw heads, enabling ease of anatomic rod contouring.

Often, iliac screws are placed by creating a breach in the iliac cortex (at the PSIS) to allow seating of the screw head, which diminishes screw purchase to only one cortex (cortical bone above the sciatic notch). Akesen *et al*.^[20]^ noted that iliac screws have a much weaker bone and screw interface than lumbar or sacral pedicle screws as a result of their positioning in cancellous bone. These authors theorized that a better sacropelvic fixation technique would diminish screw-toggling stress, which, in turn, could reduce screw loosening. This iliac-toggling stress can be reduced by the sacroiliac screw *via* its insertion through four cortices (sacral dorsum, both sacroiliac joint cortices, and the thick cortical bone above the sciatic notch).

### Indications

Indications for sacropelvic fixation are broad. Previously reported indications include long fusion to the sacrum for spinal deformity, medium- and high-grade isthmic spondylolisthesis, revision surgery, and surgery involving patients at high risk for fusion failure at the lumbosacral junction^[2^,^4^,^20^–^24]^.

### Advantages

The method proposed for sacropelvic fixation with bilateral sacroiliac screws provides several advantages over iliac screw fixation. Sacroiliac screws do not require modification of midline exposure for placement. In contrast, more lateral exposure is needed to allow visualization of the iliac entry point for iliac screws. This approach lengthens the surgical procedure, leading to increased blood loss. Sacroiliac screws are less prominent than iliac screws because of their deeper, more medial screw head position. In contrast, iliac screws frequently are prominent and can cause discomfort, prompting their removal. Iliac screws and their offset connectors have been associated with a substantial incidence of breakage in some series, although pseudoarthrosis or pain is not always reported^[2^,^6^,^20^,^25]^.

Additional advantages offered by sacroiliac screws include easier rod contouring and a simpler final construct, both of which reduce the possibility of instrumentation failure. Image guidance obviates the need for typical anatomic landmarks when screws are placed. Rather, an entry point on the dorsal sacrum aligned with ipsilateral lumbosacral screws may be used to allow easy positioning of the rod. The entry point for iliac screws, on the other hand, is typically not aligned with the lumbosacral pedicle screws. This poses an additional challenge in bending the rod, or results in an additional point of construct failure when offset connectors are used.

Although not tested, sacropelvic fixation with sacroiliac screws should have greater pullout strength than sacropelvic fixation with iliac screws because additional cortical walls are traversed. Four cortical walls are traversed by sacroiliac screws as opposed to one or two by iliac screws, depending on whether or not the cortical iliac entry point is removed in an effort to bury the iliac screw, thus avoiding its prominence. Additional pullout strength of the sacroiliac screw sacropelvic fixation construct may enhance successful bony fusion. The biomechanical study demonstrated L5–S1 stiffness in the sacroiliac construct comparable with that in the currently used S+I construct.

### Enhanced safety afforded by image guidance

Attempting sacroiliac screw placement without the use of an IGS is not recommended. Sacroiliac screw trajectory often requires that the surgeon’s hand be in contact with the skin and paraspinal muscle while a sounding probe is advanced past the sacroiliac joint and into the ilium. Tension from the paraspinal muscle mass reduces the bony tactile feedback upon which the surgeon normally depends to avoid cortical perforation. The use of image guidance dramatically decreases the risk to nearby pelvic structures, and effectively obviates the need for intraoperative radiographic verification. The iliac screws used are typically 7.5 mm in diameter and 60 to 80 mm long^[6^,^22^,^25]^. However, at least one radiologic and anatomic study has suggested that the maximal length of iliac screws can be up to 141 mm in men and 129 mm in women, and a diameter of 6 to 8 mm allows good purchase^[26]^. Image guidance allows for the placement of larger-diameter, longer screws with improved safety. Furthermore, using the IGS during preoperative planning will allow the practitioner to determine screw sizes in advance. This facilitates appropriate selection of the instrumentation set, as some systems do not include screws of longer length or greater diameter.

Two published reports have described sacroiliac screw fixation with the sacral insertion point starting at S2. In a study by Chang *et al.*^[27]^, a screw pathway from sacrum (S2) to ilium was proposed on the basis of 3D radiographic analysis of skeletally mature adolescents with normal pelves. In another study, O’Brien *et al.*^[28]^ described percutaneous placement of sacrum (S2)-iliac screws in cadaveric specimens on the basis of measurements derived from 3D CT images. However, we have come across no studies of sacroiliac screw placement that used image guidance. The proposed technique, which is not limited to S2 of the sacrum, may begin at S1.

Multiple studies using cadaveric specimens or plastic models have verified the safety and effectiveness of image-guided percutaneous placement of iliosacral screws^[10^–^11^,^14^–^15]^. Gautier *et al.*^[10]^ found that image guidance was safe for open or percutaneous screw fixation of the SI joint. Hinsche *et al.*^[11]^ reported that radiation exposure was considerably lessened, and that the major advantage was surgical guidance in four planes simultaneously. Smith *et al.*^[14]^ found that screws were placed more accurately under image guidance, although these investigators did encounter errors with all methods of screw placement.

**Fig.6:**
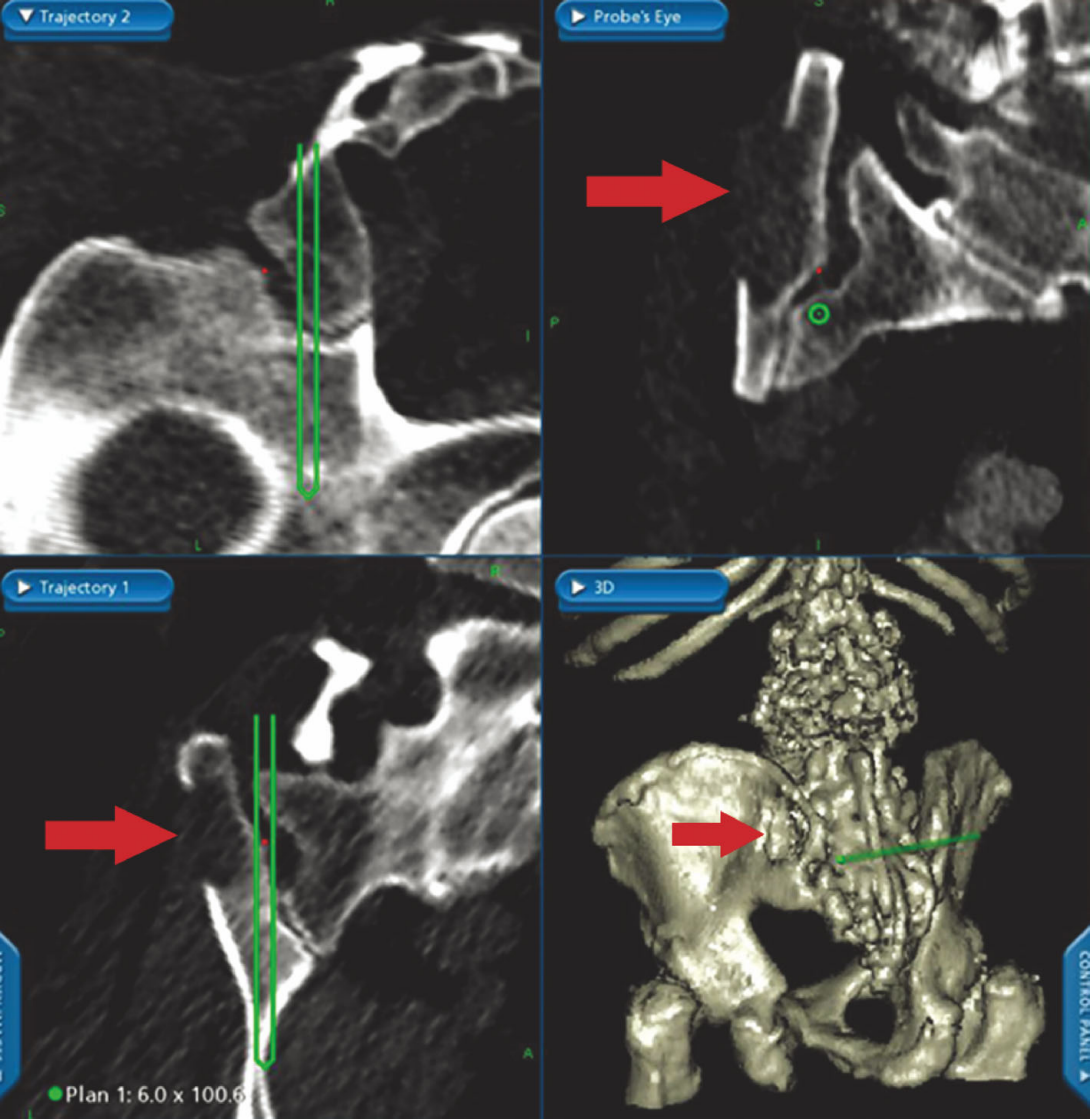
Stealth IGS snapshot taken during preoperative planning. Red arrows indicate iliac crest defect from prior graft harvest site. The planned trajectory allows for avoidance of this defect, enhancing fixation strength. IGS: image guidance system.

Tonetti *et al.*^[15]^ used four human pelves, placed all 12 screws correctly, and found the method to be safe. Inserting sacroiliac screws under IGS should not significantly lengthen operative time compared with that required for iliac screw fixation. The time consumed by registration may be balanced out by the time saved avoiding lengthy fluoroscopic imaging, which would be necessary without IGS. Furthermore, patients undergoing reoperation frequently have had a prior iliac crest graft harvest on one or both sides. With the use of IGS, the placement of sacroiliac screws in patients with prior iliac harvesting was simple. With an appropriate preoperative plan, screw insertion was effectively guided, and the previous iliac harvest site was avoided (*Fig. 6*).

### Potential complications

Potential complications can be inferred from those seen in the context of sacral pedicle screws, iliac screws and iliosacral screws, including percutaneous screws^[12^–^13^,^20]^. Practitioners may encounter iliac or sacral fracture, vascular or nerve root injury, spinal fluid leak, screw malpositioning, haloing, loosening, fatigue or failure. Technical vigilance and image guidance should minimize the occurrence of these complications. The use of sacropelvic fixation does not totally eliminate the incidence of pseudoarthrosis at L5–S1, even when anterior column support is provided^[6^,^29]^. Available literature provides no evidence that iliac screws are deleterious to the SI joint at 5 to 10 years of follow-up^[6]^.

### Limitations

Current IGS systems have limitations. The length of the sounding probe and tap is limited, as these instruments are designed for pedicle screws no longer than 50 mm. In many cases, shorter screws have had to be used, because screws longer than 70 mm were not available. Tracking the screw itself is not possible with many image guidance systems. Thus, great care must be taken to vigilantly adhere to the trajectory already established by the image-guided sounding probe and tap. Sacroiliac screw placement without image guidance may be too risky. With image guidance, most spine surgeons will be able to place sacroiliac screws successfully despite their level of expertise. However, the IGS accuracy must always be verified before navigation to avoid complications. As noted in the case of malpositioning, it is important to ensure that the preoperative CT obtained with IGS has an adequate field of view that includes both iliac crests.

In conclusions, lumbosacral fusion continues to evolve through technological advancements in spinal instrumentation, and in the development of tools that facilitate the safe and accurate placement of implants. As the U.S. demographic shifts toward a larger geriatric population, more problems with the osteoporotic spine will be encountered. Consequently, more patients will develop complex lumbosacral pathologies that will require stronger and more durable spinal fixation techniques. This new technique of image-guided sacroiliac screw insertion should prove useful in many types of fusion to the sacrum, particularly for patients with poor bone quality, complicated anatomy, infection, previous failed fusion and iliac harvesting.
